# Up-regulation of long noncoding RNA MALAT1 contributes to proliferation and metastasis in esophageal squamous cell carcinoma

**DOI:** 10.1186/s13046-015-0123-z

**Published:** 2015-01-22

**Authors:** Liwen Hu, Yuanyuan Wu, Deli Tan, Hui Meng, Kai Wang, Yun Bai, Kang Yang

**Affiliations:** Department of Cardiothoracic Surgery, Southwest Hospital, Third Military Medical University, Gaotanyan St., Shapingba District, Chongqing, People’s Republic of China; Department of Medical Genetics, College of Basic Medical Science, Third Military Medical University, Gaotanyan St., Shapingba District, Chongqing, People’s Republic of China

**Keywords:** Long noncoding RNA, MALAT1, Esophageal cancer, Copy number, Cell cycle arrest

## Abstract

**Background:**

Metastasis Associated Lung Adenocarcinoma Transcript 1 (MALAT1) has been demonstrated to be an important player in various human malignancies; it is thought to promote tumor growth by cell cycle regulating. However, the roles of MALAT1 in esophageal squamous cell carcinoma(ESCC), and the mechanisms involved in cell cycle regulation remain poorly understood. Moreover, the factors contributing to its up-regulation in tumor tissues are still largely unclear.

**Methods:**

Expression of MALAT1 was determined from cell lines and clinical samples by qRT-PCR. The effects of MALAT1 knockdown on cell proliferation, cell cycle, apoptosis, migration, and invasion were evaluated by *in vitro* and *in vivo* assays. The potential protein expression changes were investigated by Western-blotting. The methylation status of the CpG island in the MALAT1 promoter was explored by bisulfite sequencing, while the copy numbers in tumor tissues and blood samples were detected by a well-established AccuCopy^TM^ method.

**Results:**

MALAT1 was over-expressed in 46.3% of ESCC tissues, mostly in the high-stage tumor samples. Enhanced MALAT1 expression levels were positively correlated with clinical stages, primary tumor size, and lymph node metastasis. Inhibition of MALAT1 suppressed tumor proliferation *in vitro* and *in vivo*, as well as the migratory and invasive capacity. MALAT1 depletion also induced G2/M phase arrest and increased the percentage of apoptotic cells. Western-blotting results implicated that the ATM-CHK2 pathway which is associated with G2/M arrest was phosphorylated by MALAT1 knockdown. No effects of CpG island methylation status on MALAT1 expression were found, whereas amplification of MALAT1 was found in 22.2% of tumor tissues, which correlated significantly with its over-expression. However, neither association between tissue copy number amplification and germline copy number variation, nor correlation between germline copy number variation and ESCC risk were identified in the case–control study.

**Conclusions:**

Our data suggest that MALAT1 serves as an oncogene in ESCC, and it regulates ESCC growth by modifying the ATM-CHK2 pathway. Moreover, amplification of MALAT1 in tumor tissues may play an important role for its up-regulation, and it seems that the gene amplification in tumor tissues emerges during ESCC progression, but is not derived from germline origins.

**Electronic supplementary material:**

The online version of this article (doi:10.1186/s13046-015-0123-z) contains supplementary material, which is available to authorized users.

## Background

Esophageal cancer is one of the leading aggressive malignancies worldwide and ranks as one of the top five deadliest cancers in China, while squamous cell carcinoma accounts for the most prevalent histopathologic type [1]. Despite the recent advances in ESCC treatment, the prognosis of ESCC is still unfavorable [2,3], making it essential for uncovering the underlying mechanisms of ESCC for therapy improvement. The emerging roles of lncRNAs in human tumorgenesis may hold great promise for understanding the carcinogenesis of ESCC [4].

Long noncoding RNA (lncRNA) is defined as a group of RNAs (>200 nt) with limited or no coding capacity due to a lack of an intact open reading frame, but participates in most vital biological activities. LncRNAs can affect gene expression via multiple fashions, including chromatin reprogramming [5], X chromosome inactivation [6], microRNA sponging [7], and so on. A growing amount of literature has linked deregulation of lncRNAs with diverse human cancers, such as the well-characterized HOTAIR, MEG3, GAS5, H19, and MALAT1 [8].

Metastasis Associated Lung Adencarcinoma Transcript 1 (MALAT1), mapped to human chromosome 11q13, was originally identified as a prognostic marker for metastasis and patient survival in NSCLC [9]. Following studies have documented that MALAT1 was aberrantly up-regulated in multiple cancerous tissues and conferred proliferative and metastatic phenotypes to tumor cells [10-12]. It promotes tumor growth and metastasis through different mechanisms, including by recruiting SR family proteins [13], binding to the active regions of chromosome [14], and regulating alternative splicing of oncogenic mRNAs [15], depending on tissue contexts. The role that it plays in ESCC is of interest to our research group. Kuo et al. reported that exogenous over-expression of tumor suppressor gene sox17 impaired ESCC growth and mobility, with a synchronous decreased MALAT1 level [16], which indirectly implicated that MALAT1 may serve as an oncogene in ESCC.

There is a lot of evidence suggesting that MALAT1 may be involved in cell cycle regulation, which may contribute to uncontrolled tumor growth. Its role in G2/M regulation has been specifically emphasized [15,17,18], but its detailed mechanism is still controversial. Tripathi et al. reported that MALAT1 depletion leads to an increased level of γH2AX (a DNA damage indicator) and accumulated G2/M population, indicating a critical involvement of MALAT1 in genome stability and cell cycle checkpoint. To our knowledge, the tumor genomes are under frequent DNA damage events and spontaneous genetic mutation, which could activate the cell cycle checkpoints, represented by the ATM-CHK2 pathway. The activated checkpoint may prevent tumors from growing too quickly by inducing cell cycle arrest. Inactivation of this checkpoint may release cells from cycle arrest, and results in accelerating tumor growth. In consideration of the role of MALAT1 in cell cycle progression and genome stability maintenance, we speculated that MALAT1 could promote ESCC proliferation by regulating the ATM-CHK2 pathway, which is associated with DNA damage response and the G2/M transition.

Moreover, the factors that lead to MALAT1 over-expression in tumor tissues remain largely unknown, previous studies have found that the expression level of MALAT1 was controlled by methylation of histone H3 [19], transcriptional factors [16,20], and microRNAs [21,22]; however, evidence to explain its over-expression in diverse tumor tissues is lacking.

To better understand its roles in ESCC and the factors leading to its up-regulation, we investigated the clinical significance of MALAT1 on ESCC and confirmed its biological functions by *in vitro* and *in vivo* assays. We also checked the ATM-CHK2 pathway, which is involved in DNA damage response and G2/M arrest, to unravel the mechanisms by which MALAT1 regulates cell cycle progression and promotes ESCC growth. To explore the factors contributing to its up-regulation, we sequenced the CpG island located at its promoter, and detected the copy number alterations in tumor tissues. Finally, we determined whether the MALAT1 amplification in tumor tissues was derived from germline origins. We also evaluated the possibility that the germline copy number variation (CNV) of MALAT1 be used as an indicator of ESCC risk for Chinese Han people in a case–control study.

Our results showed that MALAT1 was upregulated mostly in late-stage tumor tissues, indicating that it mainly functions in the advanced stages of ESCC but not tumor initialization. Knockdown of MALAT1 suppressed proliferation and metastasis of ESCC cells, leading to G2/M arrest and an increased apoptosis ratio. MALAT1 depletion activated the ATM-CHK2 pathway, which should be responsible for G2/M arrest. Our results also revealed a negative association between MALAT1 expression and ATM-CHK2 pathway phosphorylation in tumor tissues, suggesting that up-regulation of MALAT1 may promote ESCC growth by dephosphorylation of the ATM-CHK2 pathway, which may loose the cell cycle arrest. We also found that amplification of MALAT1 in tumor tissues may partially contribute to its over-expression, but the genomic amplification in somatic tissues should be a complex event, instead of being derived from a germline source. In addition, the case–control study results indicated that the germline CNV of MALAT1 should not be treated as an indicator for ESCC susceptibility.

## Materials and methods

### Tissue samples and cell lines

ESCC and corresponding normal esophageal epithelial tissues were obtained from 54 patients who underwent surgery resection during 2007–2012 at Southwest Hospital, Chongqing, China. No patient recruited in the study received radio- or chemo-therapy prior to surgery. Clinical information was collected from medical records. All specimens were snap-frozen and stored at −80°C until use. The study was approved by the Research Ethics Committee of the Third Military Medical University, Chongqing, China. Written informed consent for biological research was obtained from all participants.

Four esophageal squamous cell carcinoma cell lines (EC109, EC9706, KYSE150, and KYSE450) were obtained from the Cell Bank of the Chinese Academy of Sciences (Shanghai, China) and Cancer Institute and Hospital, Chinese Academy of Medical Sciences (Beijing, China). A normal esophageal epithelial cell (Het-1A) was purchased from Jenniobio Biotechnology (Guangzhou, China). All cells were cultured in RPMI-1640 medium (Hyclone, USA) supplemented with 10% fetal bovine serum (10% FBS), and maintained in a humidified incubator at 37°C with 5% CO_2_.

### Case–control study population

A total of 201 cases diagnosed with ESCC and 193 ethnically-matched healthy controls were recruited from Southwest Hospital. All subjects were genetically unrelated ethnic Han-Chinese from southwest China. The protocol and consent form were approved by the Research Ethics Committee of the Third Military Medical University. All participants provided informed consent prior to enrollment.

Distribution of demographic characteristics are listed in Additional file 1: Table S1.

### RNA extraction and qRT-PCR

Total RNA was isolated from tissues and cell cultures with Trizol reagent (Takara, Japan) according to the standard protocol. The cDNA was synthesized from a total of 200 ng RNA using the PrimeScript RT reagent Kit (Takara, Japan) and amplified by quantitative real-time PCR with SYBR Green Kit (Takara, Japan) on Illumina Eco™ (Illumina, USA). GAPDH was used as the internal reference, and the relative expression level of MALAT1 was normalized to GAPDH.

Primers for MALAT1 and GAPDH are listed in Additional file 2: Table S2.

### DNA extraction

DNA was extracted from tumor tissues and blood samples using the Wizard® genomic DNA extraction Kit (Promega, USA) according to the standard procedure, the extracted genomic DNA was dissolved in DNA rehydration solution and stored at −20°C until use.

### Small interfering RNA

Two small interfering RNAs (siRNAs) against MALAT1 (si-MALAT1) at different sites and one negative control (si-NC) with no definite target were employed and synthesized by GenePharma (Shanghai, China). Cells were seeded on six-well plates at a density of 3 × 10^5^/well overnight, and then transfected with siRNA or the negative control at a final concentration of 100 nM using Lipofectamine 2000 (Invitrogen, USA). The interfering efficiency was determined by qRT-PCR 48 h after transfection, and the siRNAs with silencing efficacy of more than 70% were selected for further experiments.

Sequences of siRNAs and negative control are provided in Additional file 3: Table S3.

### Cell proliferation assay

Cells seeded on 96-well plates (5000/well) were transfected with siRNAs or NC, and the cell proliferation assays were conducted every 24 h using cell counting kit8 (Djingo, Japan) as the manufacturer’s protocol. The number of viable cells was quantified by the absorbance of reduced WST-8 at 450 nm at indicated time points.

For colony forming experiments, cells (EC109 and EC9706) transfected with siRNAs or NC were plated on six-well plates at a density of 500/well, maintained in RPMI-1640 medium containing 10% FBS for 12 days. Medium was replaced every 3 days. Colonies were fixed with methanol twelve days later and stained with 0.1% crystal violet (Sigma, USA). Visible colonies were photographed and counted manually.

### Flow-cytometric analysis of apoptosis and cell cycle

Cells seeded on six-well plates were harvested 72 h post transfection by trypsin and collected by centrifugation. Cell pellets were washed with cold PBS, and then fixed in 70% ethanol at 4°C overnight. After fixation, the cells were washed and resuspended in PBS, and then incubated with ribonuclease at 37°C for 30 minutes, next stained with propidium iodide (Beyotime, China) in the dark at 4°C for 30 minutes, and finally analyzed by a flow cytometer FACSCalibur (BD Bioscience, USA) and Modlfit software (Verity Software House, USA).

For apoptosis analysis, the trypsinized and PBS washed cells were stained with FITC-Annexin V and propidium iodide (Beyotime, China). Cells were analyzed by FACSCalibur and Flowjo software (BD Bioscience, USA). Cells were discriminated into viable cells, dead cells, early apoptotic and late apoptotic cells, relative ratios of the early and late apoptotic cells were compared to the control group.

### Transwell assay

Cell migration and invasion assays were performed using Costar chambers containing transwell inserts with a pore size of 8 μm (Corning Incorporated, USA). The upper chambers were either non-coated for migration or coated with Matrigel (Invitrogen, USA) for the invasion assay. Cells (5 × 10^4^) transfected with siRNAs or NC were suspended in 200 μl of serum-free medium and seeded in the upper chamber, while the bottom chamber contained medium mixed with 20% FBS. Thirty-six hours after incubation, cells in the upper chamber were removed by cotton swap, whereas the cells that had migrated or invaded through the membrane were fixed with methanol and stained with 0.1% crystal violet, imaged, and counted under an inverted microscope in five random fields (Olympus, Japan).

### Tumor xenografts in nude mice

Four-week athymic female BALB/c mice were housed and maintained in laminar airflow chambers under specific pathogen-free conditions. Lentiviral vectors for si-MALAT1 and NC were separately constructed by Genepharm, and the stably transfected EC109 cell lines were established. The transfected EC109 cells were suspended in PBS at a final concentration of 1 × 10^8^ cells/ml separately, a volume of 0.1 ml of suspended cells was subcutaneously injected into a single side of the posterior flank of each mouse (n = 5/group). At 21 days post injection, the mice were sacrificed by cervical dislocation in diethyl ether anesthesia, and the tumors were dissected and weighed. The experimental protocols were approved by the committee on animal experimentation of the Third Military Medical University.

### Western blotting

Cells transfected with siRNAs or NC were lysed using protein extraction reagent RIPA (Beyotime, China) 72 h post transfection, supplemented with protease inhibitors PMSF (Roche, Switzerland). ESCC tissues were ground in homogenizers and proteins were extracted following the manufacturer’s instructions (Beyotime, China). Half of the ESCC tissues showed MALAT1 over-expression and were higher than the mean expression level in ESCC, while the other half didn’t. Equal amounts of protein extractions were separated by 10% SDS-polyacrylamide gel electrophoresis (SDS-PAGE), then transferred to polyvinylidene diflvoride membranes (Sigma, USA). The membranes were blocked for 1 h at room temperature, and subsequently incubated with specific primary antibodies for 12 h. Then the membranes were washed three times and incubated with the horseradish peroxidase (HRP)-conjugated secondary antibodies (anti-rabbit) for one hour. ECL chromogenic substrate was used to visualize the bands. GAPDH was used as the control. Antibodies were all purchased from Abcam (USA).

### Methylation status analysis of CpG island

Genomic DNA prepared from ten pairs of ESCC and normal tissues was modified by sodium bisulfite using the EZ DNA Methylation Kit (Zymo Research, USA), and then amplified by PCR. PCR-amplified products were ligated to pMD18-T vectors (TAKARA, Japan) and transformed into *E.coli* DH5α cells. The LB^amp+^ agar/X-gal/IPTG plates were used to select the qualified colonies. Ten colonies for each sample were picked out and then cultured in LB^amp+^ medium overnight separately, and subsequently obtained plasmids were subjected to sequencing.

Primers for CpG island amplification are available in Additional file 2: Table S2.

### Treatment of ESCC cells with demethylase 5-aza-cdR

EC109 and EC9706 cells (3 × 10^5^) were plated on six-well plates and grown overnight. After 24 h, the media was removed and replaced with fresh media containing 0, 5, 10 μM of demethylase 5-aza-CdR (Sigma, USA). The cells treated with 5-aza-CdR were harvested 48 h post treatment and subjected to RNA extraction to determine the MALAT1 expression level. To ensure that the demethylation by 5-aza-CdR was actually occurring, the CDH1 gene which was reported to be regulated by CpG methylation in ESCC [23,24]. Primers for CDH1 are listed in Additional file 2: Table S2.

### Copy number genotyping

Copy number of MALAT1 in tumor tissues and blood samples were interrogated by a custom-by-design AccuCopy^TM^ kit (Cat#: CN0105, Genesky Biotechnologies Inc, Shanghai, China), which was developed based on a patented technology from Genesky Biotechnologies Inc. The basic principal of this method has been described previously [25], which was based on a multiplex fluorescence competitive PCR, in brief. To ensure the accuracy of copy number calculations, four reference segments were utilized for normalization. A probe targeted to MALAT1 (1500–1813 bp) was designed for MALAT1 copy number detecting. The primers for the MALAT1 probe and reference segments are provided in Additional file 2: Table S2. Our data were produced mainly according to the manufacturer’s manual.

### Statistical analysis

Each biological experiment was performed in triplicate and repeated three times. Data are shown as mean ± standard deviation (SD). Statistical significance was tested by Student’s *t*-test (two-tailed), Chi-square test and one-way ANOVA analysis as appropriate. An unconditional logistic regression model was used to assess the associations between MALAT1 copy number genotypes and ESCC risk with or without adjustment by age, gender, and smoking status. All statistical analyses were performed on SPSS 16.0. *P* < 0.05 was considered as significant.

## Results

### MALAT1 expression was up-regulated predominantly in advanced-stage ESCC tissues

**Figure 1:**
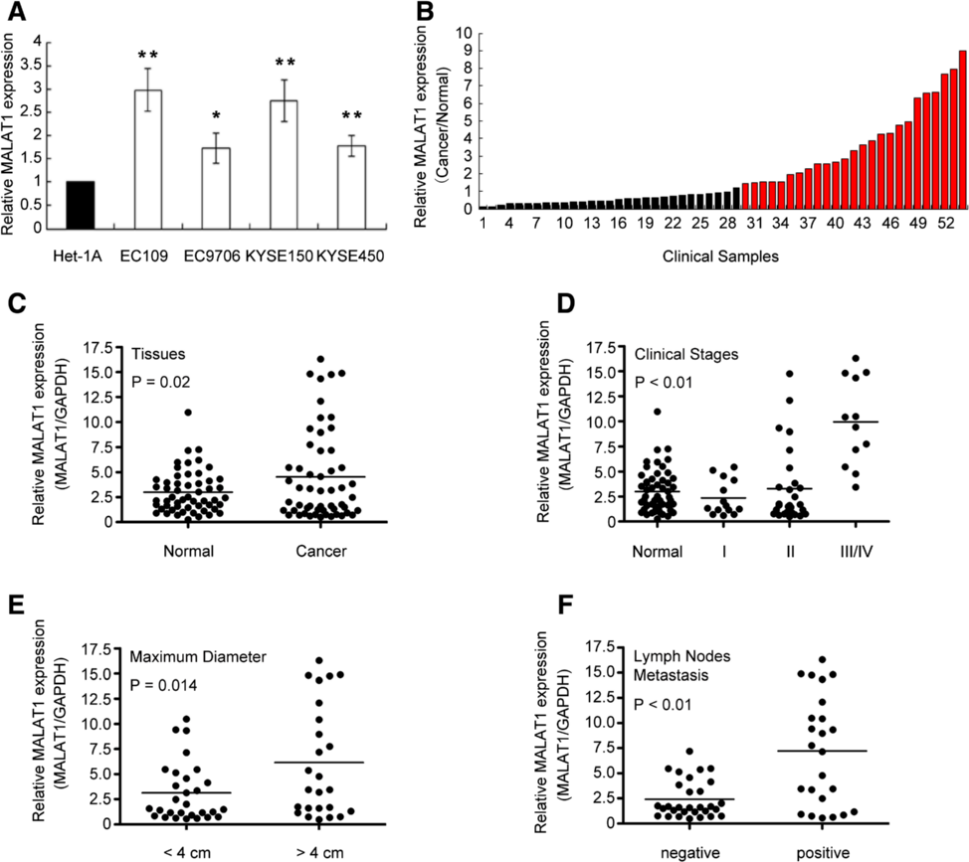
**Relative MALAT1 expression in cell lines and tissues assessed by qRT-PCR and its clinical significance. (A)** Expression level of MALAT1 in ESCC cell lines (EC109, EC9706, KYSE450, and KYSE150) compared with that of the normal epithelial cell line Het-1A, data was presented as expression fold-change relative to Het-1A. **(B)** Over-expression of MALAT1 was detected in 46.2% of ESCC tissues. Relative expression of MALAT1 was detected in 54 pairs of clinical samples and normalized to GADPH; Data was presented as fold-change in tumor tissues relative to normal tissues. The red column was defined as over-expression. **(C)** Difference in expression levels of MALAT1 between cancerous and normal esophageal tissues. The statistical differences between the two groups were analyzed by paired Student’s *t*-test. **(D)** MALAT1 expression was significantly higher in patients at advanced pathological stages; one-way ANOVA analysis was performed to calculate the statistical difference. **(E, F)** MALAT1 expression was significantly higher in samples with larger tumor size and lymph node metastasis. The statistical differences between the two groups were analyzed using unpaired Student’s *t*-test. *P < 0.05, **P < 0.01.

qRT-PCR was used to detect MALAT1 expression levels in cell lines and clinical samples, which were normalized to GAPDH. All the four ESCC cell lines expressed higher MALAT1 than the normal cell line, especially in EC109 and KYSE150 (Figure 1A).

The results from tissues showed that MALAT1 was up-regulated in 46.3% (25/54, Figure 1B) of ESCC tissues (cancer/normal ratio > 1.5), with the mean expression level of 4.53 in tumors and 3.02 in their counterparts (p = 0.02, Figure 1C). Furthermore, correlation analysis of MALAT1 expression with clinicopathological parameters revealed that MALAT1 was predominantly up-regulated in late-stage but not early-stage tumor tissues (Figure 1D), which indicated that MALAT1 may play its oncogenic role mostly in ESCC with advancing progression but not in the initial phase. Additionally, we found a positive association of MALAT1 expression with tumor size and lymph nodes metastasis (Figure 1E and F).

We next classified the 54 ESCC patients into two groups according to MALAT1 expression level (over-expression or not). Clinical pathologic factors were compared between the two groups (Table 1). The over-expression group was correlated with larger tumor size (P = 0.014) and lymph nodes metastasis (P < 0.01). However, the relative MALAT1 expression level was not associated with other parameters such as gender (P = 0.651), age (P = 0.984), nor tumor differentiation (P = 0.991).

**Table 1:** **Correlation between MALAT1 expression and clinicopathological parameters of ESCC**

		**Expression pattern**		
**Clinicopathological parameters**		**Non-overexpression**	**Overexpression**	***p*** **-value** ^**a**^
		n = 29	n = 25	
Gender	Male	18	14	0.651
	Female	11	11	
Age	<60	15	13	0.984
	>60	14	12	
Differentiation	Well	7	6	0.991
	Moderate/Poor	22	19	
Metastasis	Negative	21	9	0.007^b^
	Positive	8	16	
Maxium diameter	≤4.0 cm	19	8	0.014^b^
	>4.0 cm	10	17	

^a^Chi-squared test results.

^b^Significant difference.

### Knock down of MALAT1 in ESCC cells decreased cell growth *in vitro* and *in vivo*

**Figure 2:**
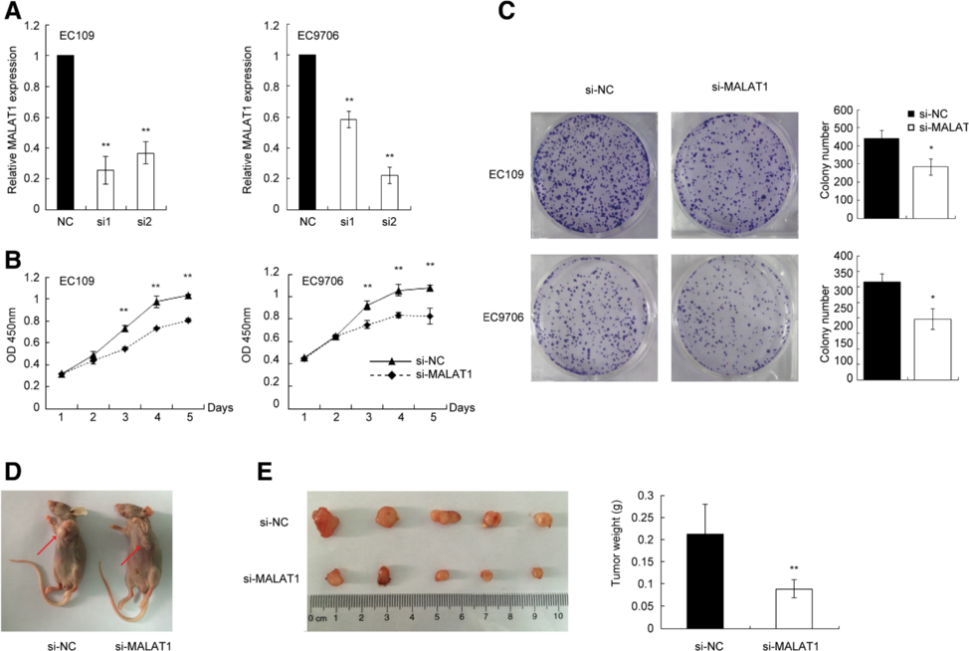
**Knockdown of MALAT1 inhibits esophageal cancer cells growth** ***in vitro*** **and** ***in vivo*** **. (A)** Expression level of MALAT1 in ESCC cell lines following treatment with si-MALAT1 or si-NC. si1 and si2 indicate the two small interfering RNA used in this study. **(B)** Knockdown of MALAT1 suppressed ESCC growth *in vitro*. CCK8 assays were performed to determine the proliferation of ESCC cells transfected with siMALAT1 and si-NC. **(C)** Colony-forming growth assays were also performed to determine the proliferation of EC109 and EC9706 cells. The colonies were counted and captured. **(D)** Representative image of tumor growth on nude mice. **(E)** Effects of MALAT1 knockdown on tumor growth *in vivo*, tumors developed from si-MALAT1 stably transfected EC109 cells showed smaller tumor volume and weights. *0.05, **P < 0.01.

Due to the abnormal expression of MALAT1 in ESCC cell lines and tissues, we speculated that it might have some potential roles in ESCC progression. To assess the role of MALAT1 in ESCC growth, we first silenced MALAT1 expression in EC109 and EC9706 by small interfering RNA. The siRNA which decreased MALAT1 expression level by more than 70% was chosen for further experiments. Of these two siRNAs, siRNA1 was chosen for further assays of EC109 while siRNA2 was selected for EC9706 (Figure 2A).

CCK8 assays revealed that cell growth was suppressed in both cell lines transfected with siRNAs compared with negative control (Figure 2B), colony-formation ability was also reduced by MALAT1 silencing in EC9706 and EC109 cells (Figure 2C). The inhibitory effect of MALAT1 knockdown on ESCC proliferation was also observed in a nude mice tumor growth model. Growth of tumors from the MALAT1-depleted xenografts was significantly inhibited compared with the control group (Figure 2D and E).

### Knockdown of MALAT1 impaired cell migration and invasion

**Figure 3:**
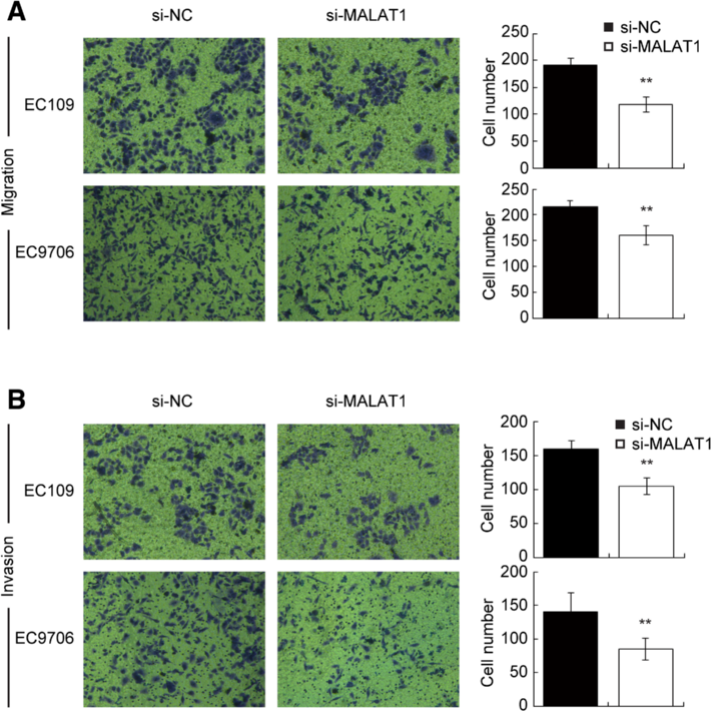
**Inhibition of MALAT1 impaired invasive and migratory capacity of esophageal cancer cells.** Representative images of the transwell migration **(A)** and invasion **(B)** assays, the bar chart represented the number of cells migrated or invaded into the lower chambers; Magnification: 100×. *P < 0.05, **P < 0.01.

We investigated cancer cells migratory and invasive capacity through transwell assays. Compared with control groups, MALAT1-deficient cells displayed impeded migration in both EC109 and EC9706 cells. The migration decreased by approximately 38.2% and 25.6% respectively (Figure 3A). Similarly, invasion of EC109 and EC9706 cells was reduced by 34.2% and 39.8% separately following silencing of MALAT1 (Figure 3B).

### Knockdown of MALAT1 induced cell cycle arrest by activation of the ATM-CHK2 pathway

**Figure 4:**
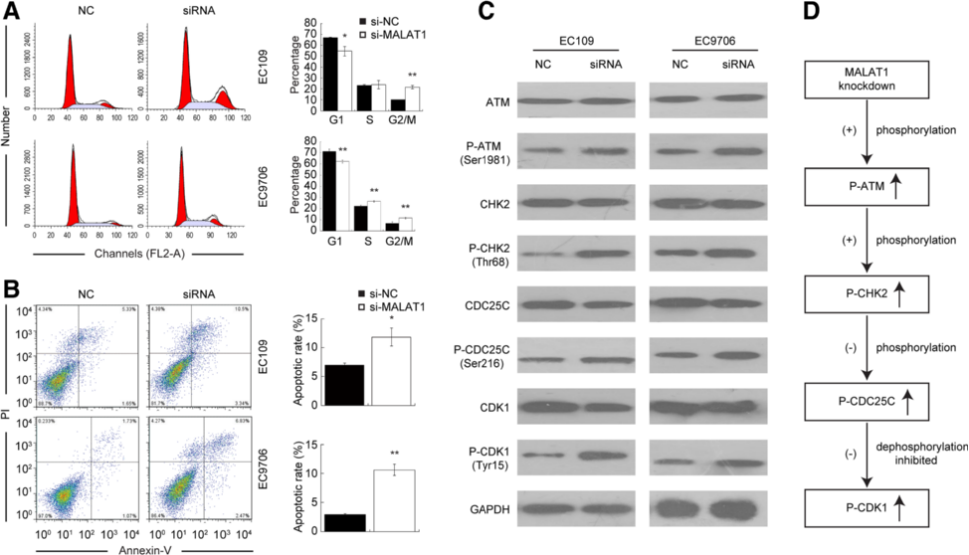
**Effects of MALAT1 depletion on cell cycle and apoptosis reflected by flow cytometry and the possible mechanism for cell cycle arrest. (A)** Representative images of cell cycle distribution, the bar chart represented the percentage of cells in G0/G1, S, or G2/M phases, as indicated. **(B)** Representative images of cell apoptosis determined by flow cytometry analysis, the bar chart represented the sum percentage of early and late apoptotic cells. **(C)** Western-blotting analysis of the ATM-CHK2 pathway in EC109 and EC9706 cells; The phosphorylated forms of ATM, CHK2, CDC25C, and CDK1 were up-regulated upon MALAT1 knockdown, while the total amounts of these proteins exhibited no detectable changes. **(D)** The putative mechanism of MALAT1 knockdown induced cell cycle arrest in G2/M on human esophageal cancer cells. ATM is activated by phosphorylation upon MALAT1 knockdown. This activation possibly increases the level of P-CHK2 by phosphorylation at Thr68 of CHK2, while the activated P-CHK2 further inactivates CDC25C by phosphorylation at Ser216 of CDC25C. As a result, the dephosphorylation capacity of P-CDC25C on P-CDK1 (Tyr15) is inhibited, so the inactivated form of CDK1 accumulates, which contributes to G2/M phase arrest. (+): activation, (−): inactivation. *P < 0.05, **P < 0.01.

Given that MALAT1 obviously promotes ESCC cell growth *in vitro* and *in vivo*, we next determined whether MALAT1 has any impact on cell cycle and apoptosis. In both cell lines, a significantly increased proportion of G2/M phase cells were observed among MALAT1 knockdown cells compared with that of control groups (Figure 4A). The apoptotic rate of EC109 and EC9706 cells transfected with siRNAs was also elevated (Figure 4B).

Further exploration of the possible mechanisms involved in the cell cycle arrest was performed by Western-blotting. For G2/M arrest is usually induced by increased DNA damage or genome instability, we proposed that the pathway responding to chromosome lesions may be activated by MALAT1 depletion. The results showed that the expression level of phosphorylated ATM, CHK2, CDC25C, and CDK1 were significantly increased upon MALAT1 depletion, while no detectable differences were observed for total ATM, CHK2, CDC25C, and CDK1 (Figure 4C). These data suggested that inhibition of MALAT1 may activate the ATM-CHK2 pathway, and eventually lead to G2/M arrest.

**Figure 5:**
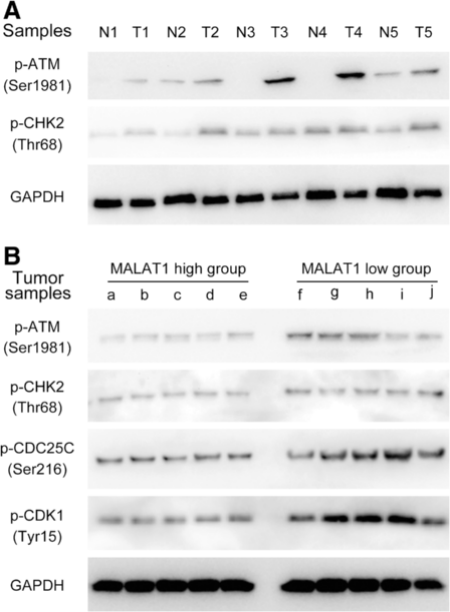
**The phosphorylaion of ATM-CHK2 pathway in tissues reflected by Western-blotting. (A)** Phosphorylaion forms of ATM and CHK2 in five pairs of cancerous and normal tissues; N: Normal tissues; T: Tumor tissues. **(B)** Phosphorylaion of ATM-CHK2 pathway in MALAT1 high-expression and low-expression groups.

Then we detected the phosphorylated forms of ATM and CHK2 in five pairs of tissues, and the results showed that the phosphorylaion of ATM and CHK2 is increased in tumor tissues (Figure 5A), suggesting that the DNA damage events may actually occur in ESCC tissues, which can activate the ATM-CHK2 pathway and prevent tumor cells from growing too quickly.

To further confirm the association between MALAT1 and the ATM-CHK2 pathway, we examined the phosphorylated forms of ATM, CHK2, CDC25C, and CDK1 in ten ESCC tissues. Samples with high MALAT1 level (> mean value, and over-expressed in ESCC) showed relatively lower levels of ATM-CHK2 pathway phosphorylation (Figure 5B), suggesting a negative correlation between MALAT1 expression and phosphorylation of the ATM-CHK2 pathway in ESCC tissues, which is consistent with results from cell lines. Together these data suggested that high expression of MALAT1 promotes ESCC proliferation by dephosphorylation of the ATM-CHK2 pathway, which may release cells from G2/M arrest, resulting in uncontrolled cell cycle and tumor growth.

### Amplification of MALAT1 in tumor tissues may partially contribute to its over-expression

**Figure 6:**
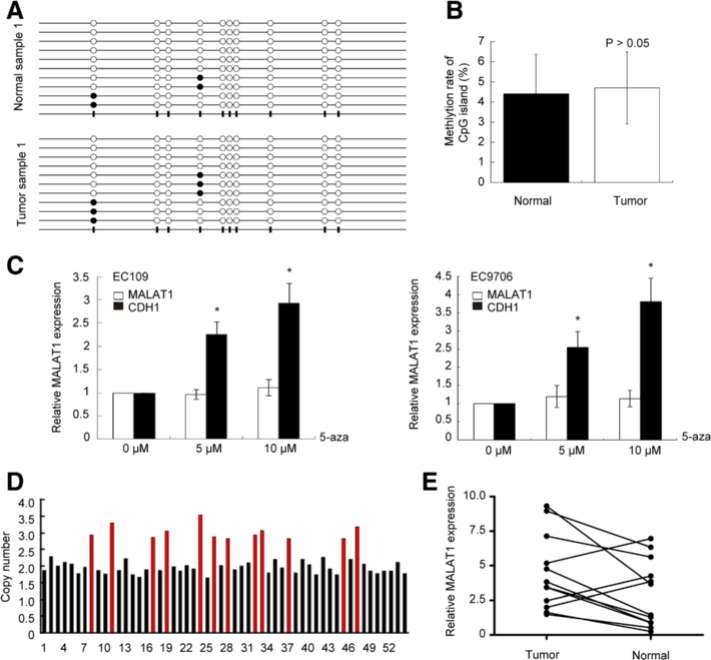
**Effects of CpG island methylation status and copy number amplification on MALAT1 expression level. (A)** Methylation status of the CpG island in a representative pair of clinical tissues, assessed by bisulfite sequencing. Open and filled squares denote un-methylated and methylated CpG sites respectively; each row represents a single clone. **(B)** No obvious difference in CpG island methylation frequency was observed in the ten pairs of tissues. A paired Student’s *t*-test was performed to determine the statistical significance. **(C)** The expression level of MALAT1 and CDH1 in EC109 and EC9706 cells following treatment with demethylase agent 5-aza-cdR (0, 5, and 10 μM). **(D)** Genomic copy number of MALAT1 in tumor tissues calculated by AccuCopy™ system. Red bars represented samples with MALAT1 amplification in tumor tissues. **(E)** Relative expression level of MALAT1 (normalized to GAPDH) in the twelve pairs of tissues, which showed gene amplification in tumor tissues. *P < 0.05, **P < 0.01.

To explore the underlying mechanisms contributing to MALAT1 up-regulation in ESCC tissues, we first investigated the methylation status of the CpG island at the MALAT1 promoter in ten paired tissues by bisulfite sequencing. We sequenced the site of the 269 bp-430 bp of the CpG island, and the results revealed the total hypomethylation rate in both tumor and normal tissues, with the average frequency of methylation merely 4.4% in normal tissues and 4.7% in ESCC tissues (Figure 6A and B). We then treated EC109 and EC9706 cells with DNA demethylase agent (5-aza-CdR), and the results showed that CDH1 was up-regulated in agent treated ells, suggesting that demethylation by 5-aza-CdR is effective. However, MALAT1 expression was not significantly changed in demethylase treated cells (Figure 6C). These data supported the idea that there were no obvious effects of CpG island methylation status on MALAT1 expression.

We next detected the copy number of MALAT1 in the 54 tumor tissues by AccuCopy™, for MALAT1 is located on human chromosome 11q13, which is reported to be frequently amplified in ESCC tissues. Amplification of MALAT1 was found in 12 tumor tissues (Figure 6D), among which nine showed increased MALAT1 expression over their counterparts (Figure 6E), while lack of over-expression in tumor tissues without amplification was observed in 48.1% of the cases, suggesting significant correlation between MALAT1 over-expression and its amplification (Table 2). These results indicated that up-regulation of MALAT1 observed in ESCC tissues might be partially due to amplification of the MALAT1 genomic region.

**Table 2:** **Association between MALAT1 amplification and its over-expression in ESCC tissues**

		**Expression level**		***p*** **–value** ^**a**^
		**Over-expression**	**Non over-expression**	
Copy number status in tumor tissues	Amplification	9	3	0.046^**b**^
	Non-amplification	16	26	

^a^Fisher’s exact test.

^b^Significant difference.

### No association of germline copy number variation with somatic copy number amplification or ESCC risk was found in the case–control study

According to Database of Genomic Variation, both germline copy number gains and losses within the MALAT1 locus were recurrently identified by multiple research groups (Additional file 4: Figure S1A). Here we detected whether the germline copy number variation of MALAT1 may lead to tissue copy number amplification and is associated with ESCC risk in a case–control study, which contained blood samples from the 54 patients mentioned above. The probe for MALAT1 copy number detection was designed to overlap with the recorded CNV Variation_65973 within the MALAT1 genominc region (Additional file 4: Figure S1A). Finally we found no germline copy number gain in whole samples while copy number loss in twelve patients and eight controls (Additional file 4: Figure S1B), suggesting that the amplification in tissues may not come from germline source, and the germline CNV should not be a risk indicator for ESCC (Table 3).

**Table 3:** **No association of germline copy number variation with ESCC risk was found in the case–control study**

**genotypes**	**No. of cases**	**No. of controls**	**Crude OR (95%CI)**	***p*** **-value**	**Adjusted OR (95%CI)** ^**b**^	***p*** **-value**
+/+^**a**^	189	185	1.00 (reference)		1.00 (reference)	
+/−^**a**^	12	8	1.437 (0.574–3.596)	0.438	1.385 (0.544–3.522)	0.494

^a^Genotypes: +/+, wild-type; +/−, hemizygous deletion.

^b^Adjusted by gender, age and smoking status.

## Discussion

Roles of lncRNAs in carcinogenesis have attracted more attention than ever before, as recent studies have found that lncRNAs are closely associated with tumor growth, epithelial-mesenchymal transition, and metastasis. However, little is known about their roles in ESCC. Previously researchers have discovered that the lncRNAs HOTAIR, POU3F3 and SPRY4-IT1 promoted ESCC progression and may be used as prognostic biomarkers, suggesting the critical roles of lncRNAs in ESCC [26-28].

Among these emerging functional stars, MALAT1 has been widely accepted as a key regulator in development and carcinogenesis, which participates in biological events through various mechanisms, such as chromosome modification [13], mediating mRNA relocation [29], and alternative splicing [15,17,30]. Recent studies have confirmed its oncogenic role in multiple cancer types, such as bladder, breast, and prostate cancers [31-33]. However, no studies on how it functions in human ESCC have been performed until now. Here we characterized its roles in ESCC for the first time.

First, we measured the expression level of MALAT1 in cell lines and clinical tissues by qRT-PCR. Over-expression of MALAT1 was found in 46.3% of ESCC tissues, mostly in advanced tumor tissues with larger tumor size and lymph nodes metastasis, indicating that MALAT1 may primarily participate in ESCC advancing but not initialization, which may be a promising biomarker for late-stage ESCC with metastasis. Inhibition of MALAT1 suppressed tumor growth *in vitro* and *in vivo*, as well as cell migratory and invasive capacity, confirming its oncogenic roles in ESCC.

Further results suggested that knockdown of MALAT1 inhibited ESCC cell proliferation by inducing G2/M stage arrest and increasing the apoptosis ratio. The role of MALAT1 in G2/M transition regulation has been previously recognized, but the underlying mechanisms remain largely elusive. Yang et al. hypothesized that MALAT1 interacted with nuclear protein hnRNP C and facilitated its cytoplasmic translocation in the G2/M phase [18], thereby regulating the progression of the cell cycle through hnRNP C. Meanwhile, Tripathi et al. reported that MALAT1-depleted cells displayed reduced expression of B-MYB, an oncogenic transcriptional factor involved in G2/M progression, resulting in cell cycle arrest [17]. Tripathi *et al.* also found increased DNA damage occurred in MALAT1 depleted cells, which should be a triggering signal for G2/M arrest. DNA damage is a common event which occurs in tumor progression, induced by both the tumor microenvironment and genomic instability. The ATM-CHK2 pathway is an important component of cell cycle regulation, which responds to DNA damage signals, and prevents tumors growing too fast by arresting cell cycle in G2/M stage [34]. Here we found inactivation of ATM-CHK2 pathway by MALAT1 over-expression for the first time.

Results from Western-blotting showed that the phosphorylated forms of ATM, CHK2, CDC25C, and CDK1 were upregulated upon MALAT1 knockdown. As we know, ATM is a key regulator in cell cycle moderating, which may be phosphorylated at Ser1981 in response to environmental disturbance and genome instability, such as toxicant-induced or spontaneous DNA damage [35-37]. The phosphorylated form of ATM could subsequently activate CHK2 by phosphorylating CHK2 at the Thr68 site [38], and the activated CHK2 played its role in suppressing CDC25C by phosphorylation on Ser216 of CDC25C [39]. The CDC25C was a phosphatase which can direct dephosphorylation of P-CDK1 (Thr14, Tyr15) and triggers entry into mitosis. When phosphorylated, the P-CDC25C showed a reduced ability to dephosphorylate P-CDK1, which was reported to be an inactive form of CDK1, preventing cell cycle progression [40]. As a result, P-CDK1 accumulation occurred and the cell cycle was arrested in G2/M (Figure 4D).

Results from ESCC tissues further supported the idea that MALAT1 promotes tumor growth by inactivating the cell cycle checkpoint. In ESCC tissues, MALAT1 expression was negatively associated with phosphorylation of the ATM-CHK2 pathway, suggesting over-expression of MALAT1 may contribute to rapid tumor growth by suppressing cell cycle arrest. In total, our results highlighted the importance of MALAT1 in ATM-CHK2 pathway regulation, which may help us to interpret the mechanisms on how MALAT1 regulates tumor growth from a new perspective. However, more detailed information about the association between MALAT1 expression and ATM-CHK2 pathway is still required.

Although over-expression of MALAT1 has been reported in multiple cancer types, little is known about the factors which contribute to its up-regulation. Here we sought to explore the uncharted factors contributing to its up-regulation. Methylation of the CpG island in the promoter may be a crucial mechanism in regulating gene expression on an epigenetic level [41]. By computationally screening the promoter of MALAT1, we found a CpG island which is 441 bp long. As no previous studies revealed whether this CpG island was associated with MALAT1 expression, we checked the correlation between the methylation status of the CpG island and MALAT1 expression level by bisulfite sequencing. The total methylation rate in both groups were extremely low and showed no apparent differences, indicating that the methylation status of MALAT1 promoter may not be a major factor contributing to its deregulation. No obvious changes in MALAT1 expression level upon treatment of ESCC cells with demethylase 5-aza-cdR further supported this idea.

MALAT1 is located on 11q13 of the human genome, which has been verified to be frequently amplified in ESCC tissues [42,43], and recent studies have discovered the tight correlation between lncRNAs amplification with their expression level [44,45], we then detected the genomic copy number of MALAT1 in the 54 tumor tissues mentioned above by a newly developed method AccuCopy™, which has been applied in other excellent works, and proven to be efficient and accurate [25,46]. The results showed that amplification of MALAT1 was found in 12 ESCC tissues, among which nine showed an over-expression pattern, implicating that the up-regulation of MALAT1 in tumor tissues may partially be attributed to copy number amplification in somatic tissues.

As amplification of MALAT1 was found in ESCC tissues, then we wondered how the amplification in tumor tissues occurred. Previous studies have proclaimed that the germline copy number variation may be an important source of somatic genome abnormality [46,47], and germline copy number gains across MALAT1 have been reported by other groups [48,49]. We therefore tested if this association also existed in the MALAT1 region. In this study, we genotyped the copy number of MALAT1 in 201 ESCC patients (the 54 patients mentioned above included) and 193 healthy controls. Surprisingly, we found no copy number gain in germline samples, indicating that the CNV status may differ in diverse ethnic groups, and it is hard to predict whether the amplification of MALAT1 in tumor tissues came from the germline origin. So we believe that the amplification of MALAT1 in ESCC tissues may be a complicated event arising during the progression phase of ESCC, but not being simply derived from germline origin. Moreover, we did not find any frequency discrepancy of germline copy number loss between the cases and controls groups, indicating that the germline copy number variation on MALAT1 should not be treated as an ESCC susceptibility indicator, in spite of its significant role in ESCC.

## Conclusions

Together these findings indicate that MALAT1 is a player of great importance in ESCC. It may promote ESCC growth by moderating the ATM-CHK2 pathway which is involved in cell cycle orchestrating, and the amplification of MALAT1 may be an important cause for its up-regulation in ESCC tissues. However, much more work is still required to determine the detailed mechanisms it functions in ESCC and the potentiality of MALAT1 as a therapeutic target for ESCC.

## Additional files

Additional file 1: Table S1. Distribution of demographic characteristics for cases and controls. Additional file 2: Table S2. Primers used in this study. Additional file 3: Table S3. Sequences of the small interfering RNAs and negative control. Additional file 4: Figure S1. No difference in copy number variation between the cases and controls groups was found. **(A)** Copy number variations across MALAT1 were identified by multiple groups; Red: Copy number losses; Blue: copy number gains. **(B)** Copy numbers of MALAT1 in the case–control population calculated by AccuCopy™. Each bar represents the copy number for its carrier; CA: cases; CTR: controls.

## Abbreviations

ESCC: Esophageal squamous cell carcinoma
NSCLC: Non-small cell lung cancer
CNV: Copy number variation
lncRNA: Long noncoding RNA
cDNA: Complementary DNA
qRT-PCR: Quantitative Reverse Transcription-PCR
HOTAIR: HOX Transcript Antisense RNA
MEG3: Maternally expressed 3
GAS5: Growth arrest-specific 5
MALAT1: Metastasis associated lung adenocarcinoma transcript 1
LB: Lysogeny broth
X-gal: 5-Bromo-4-chloro-3-indolyl β-D-galactopyranoside
IPTG: Isopropyl β-D-1-Thiogalactopyranoside
ATM: Ataxia telangiectasia-mutated kinase
CHK2: Checkpoint kinase 2
CDC25C: Cell division cycle 25C
CDK1: Cyclin-dependent kinase 1
CDH1: Cadherin 1
GAPDH: Glyceraldehyde-3-phosphate dehydrogenase
