# Supplementary material for: Up-regulation of long noncoding RNA MALAT1 contributes to proliferation and metastasis in esophageal squamous cell carcinoma
Source: J Exp Clin Cancer Res. 2015 Jan 22;34(1):7. doi: 10.1186/s13046-015-0123-z (PMC4322446; doi:10.1186/s13046-015-0123-z)
Supplement: Additional file 1: Table S1. — Distribution of demographic characteristics for cases and controls. [file 13046_2015_123_MOESM1_ESM.doc]

**Table S1 Distribution of demographic characteristics for cases and controls**

|  |  | **No. of cases** | **No. of controls** | **P-value ^a^** |
| --- | --- | --- | --- | --- |
| **Sex** | **Male** | 142 | 126 | 0.254 |
|  | **Female** | 59 | 67 |  |
|  |  |  |  |  |
| **Age** | **<40** | 2 | 6 | 0.414 |
|  | **40-50** | 31 | 36 |  |
|  | **50-60** | 124 | 118 |  |
|  | **60-70** | 39 | 29 |  |
|  | **>70** | 5 | 4 |  |
|  |  |  |  |  |
| **Smoking** | **Never** | 109 | 134 | 0.002 **^b^** |
|  | **Ever** | 92 | 59 |  |
|  |  |  |  |  |
| **Stage** | **I** | 28 |  |  |
|  | **II** | 127 |  |  |
|  | **III** | 39 |  |  |
|  | **IV** | 7 |  |  |

**^a^** Chi-squared test results.

**^b^** Significant difference
