# Supplementary material for: Up-regulation of long noncoding RNA MALAT1 contributes to proliferation and metastasis in esophageal squamous cell carcinoma
Source: J Exp Clin Cancer Res. 2015 Jan 22;34(1):7. doi: 10.1186/s13046-015-0123-z (PMC4322446; doi:10.1186/s13046-015-0123-z)
Supplement: Additional file 2: Table S2. — Primers used in this study. [file 13046_2015_123_MOESM2_ESM.doc]

**Table S2 Primers used in this study**

|  | Names | Sequences |
| --- | --- | --- |
| qRT-PCR primers | MALAT1-forward | GAATTGCGTCATTTAAAGCCTAGTT |
|  | MALAT1-reverse | GTTTCATCCTACCACTCCCAATTAAT |
|  | CDH1-forward | TGGACCGAGAGAGTTTCCCT |
|  | CDH1-reverse | ACGACGTTAGCCTCGTTCTC |
|  | GAPDH-forward | TGTTGCCATCAATGACCCCTT |
|  | GAPDH-forward | CTCCACGACGTACTCAGCG |
| Accucopy primers | MALAT1-forward | AGCATGAGGAAGGAAAAGATAAAAGG |
|  | MALAT1-reverse | ACCTTGAAATCCATGACGCAGG |
|  | Reference1- forward **^a^** | TGAGCCAAAAATTCAGAATACAAGGA |
|  | Reference1- reverse **^a^** | GTTTGCCTGCCTTCCAAGCAA |
|  | Reference2- forward **^b^** | CACTGAGCCCCAGAGACCTGAC |
|  | Reference2- reverse **^b^** | GTTTTCCCTGGAGGTGTGCATT |
|  | Reference3- forward **^c^** | AGGGTGCTGGGATCAGAGAGAG |
|  | Reference3- reverse **^c^** | CATTTTGCCACCCTCCAGTAGC |
|  | Reference4- forward **^d^** | TCCTCCACCAAGCTGATGTGTT |
|  | Reference4- reverse **^d^** | CTATTTCGGGGACAGGCCTGAA |
| CpG island primers | MALAT1-CpG-forward | GATTTTTGTAAAGGATTGGGGTT |
|  | MALAT1-CpG-reverse | CCCAAAAACTCTAAAAAACCTAAACT |

The target sequences for reference primers are located on 2p (a), 10pL (b), 20q (c), 16p (d) separately
