# Supplementary material for: Up-regulation of long noncoding RNA MALAT1 contributes to proliferation and metastasis in esophageal squamous cell carcinoma
Source: J Exp Clin Cancer Res. 2015 Jan 22;34(1):7. doi: 10.1186/s13046-015-0123-z (PMC4322446; doi:10.1186/s13046-015-0123-z)
Supplement: Additional file 3: Table S3. — Sequences of the small interfering RNAs and negative control. [file 13046_2015_123_MOESM3_ESM.doc]

**Table S3 Small interfering RNA sequences and negative control**

| Names | Sequnces |
| --- | --- |
| siRNA1-MALAT1 | GAGGUGUAAAGGGAUUUAUTT |
| siRNA2-MALAT1 | CCCUCUAAAUAAGGAAUAATT |
| Negative control | UUCUCCGAACGUGUCACGUTT |
