# Supplementary figures and images for: Up-regulation of long noncoding RNA MALAT1 contributes to proliferation and metastasis in esophageal squamous cell carcinoma
Source: J Exp Clin Cancer Res. 2015 Jan 22;34(1):7. doi: 10.1186/s13046-015-0123-z (PMC4322446; doi:10.1186/s13046-015-0123-z)

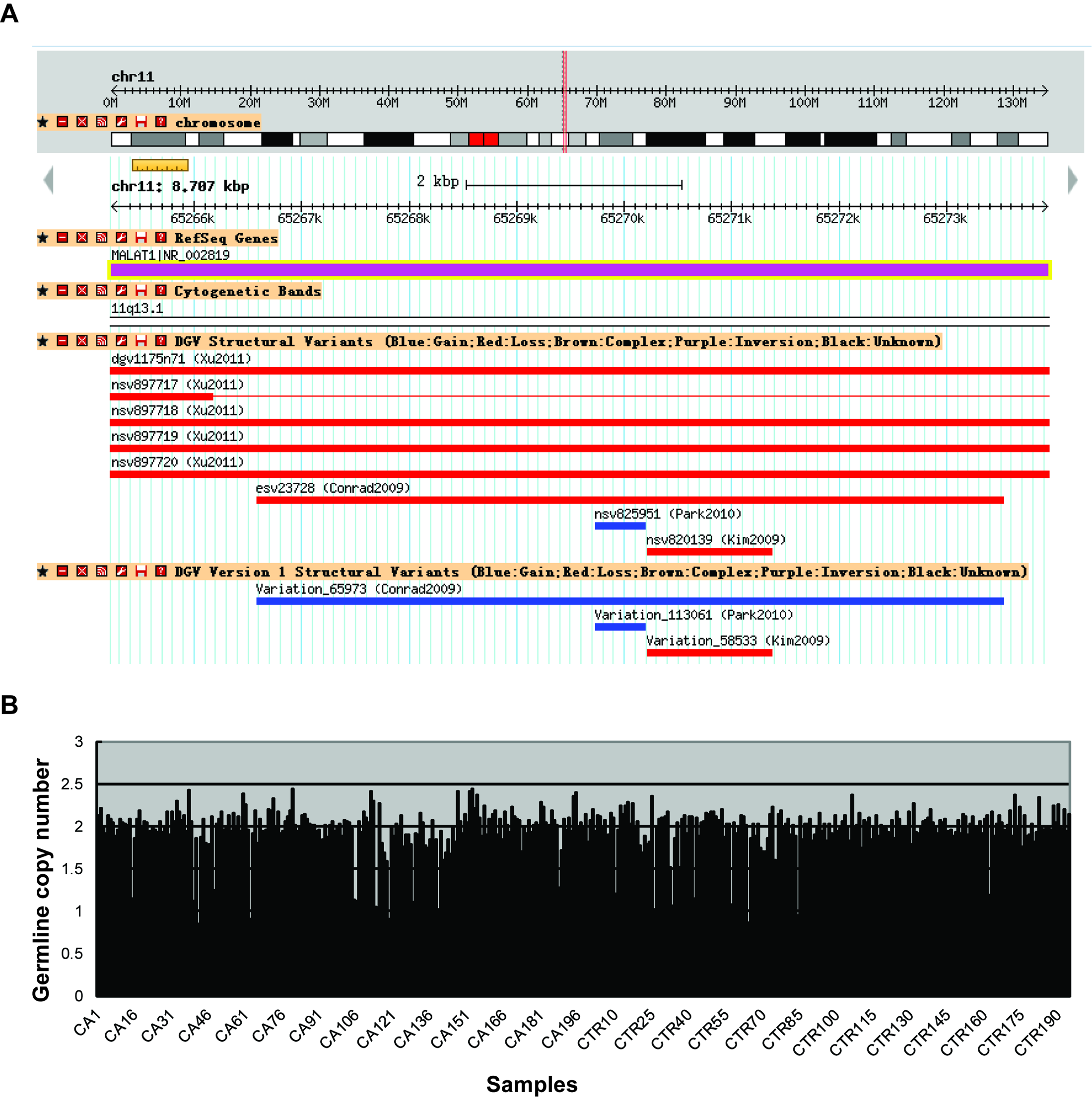

Supplement: Additional file 4: Figure S1. — No difference in copy number variation between the cases and controls groups was found. (A) Copy number variations across MALAT1 were identified by multiple groups; Red: Copy number losses; Blue: copy number gains. (B) Copy numbers of MALAT1 in the case–control population calculated by AccuCopy™. Each bar represents the copy number for its carrier; CA: cases; CTR: controls. [file 13046_2015_123_MOESM4_ESM.tiff]
